# High‐Temperature Magnetic Field Mapping Using a Single‐Crystal Diamond MEMS Resonator Array

**DOI:** 10.1002/advs.77779

**Published:** 2026-09-16

**Authors:** Zilong Zhang, Keyun Gu, Satoshi Koizumi, Masaya Toda, Meiyong Liao

**Affiliations:** ^1^ Research Center for Electronic and Optical Materials National Institute for Materials Science (NIMS) Tsukuba Ibaraki Japan; ^2^ Institute of Metal Research Chinese Academy of Sciences Shenyang China; ^3^ Graduate School of Engineering Tohoku University Sendai Miyagi Japan; ^4^ University of Tsukuba Tsukuba Ibaraki Japan

**Keywords:** array, high temperature, magnetic field imaging, microresonator, single‐crystal diamond

## Abstract

Magnetic imaging has long faced fundamental trade‐offs among speed, sensitivity, and spatial resolution, and its performance typically deteriorates at elevated temperatures. Here, we present a single‐crystal diamond (SCD) microelectromechanical system (MEMS) resonator array for spatial magnetic‐field mapping at elevated temperatures. An 8 × 8 cantilevers array enables pitch‐limited spatial magnetic‐field mapping with millisecond single‐pixel temporal response and nT Hz^1/2^ magnetic noise level at 573 K, while maintaining frequency stability at parts‐per‐billion precision. These capabilities exhibit competitive performance in sensing metrics, enabling two‐ and three‐dimensional magnetic‐field reconstruction and demonstrate elevated‐temperature magnetic‐field mapping up to 573 K. These results demonstrate the potential of SCD MEMS resonator array for spatial magnetic‐field sensing under elevated‐temperature conditions.

## Graphical abstract

A single‐crystal diamond MEMS resonator array enables magnetic‐field reconstruction with millisecond response, nanotesla level magnetic noise, and stable operation up to 573 K. Two‐ and three‐dimensional magnetic field mapping are demonstrated using Δ*E*effect sensing, highlighting the potential of diamond MEMS technology for hightemperature magnetic sensing and spatial magnetic field imaging.

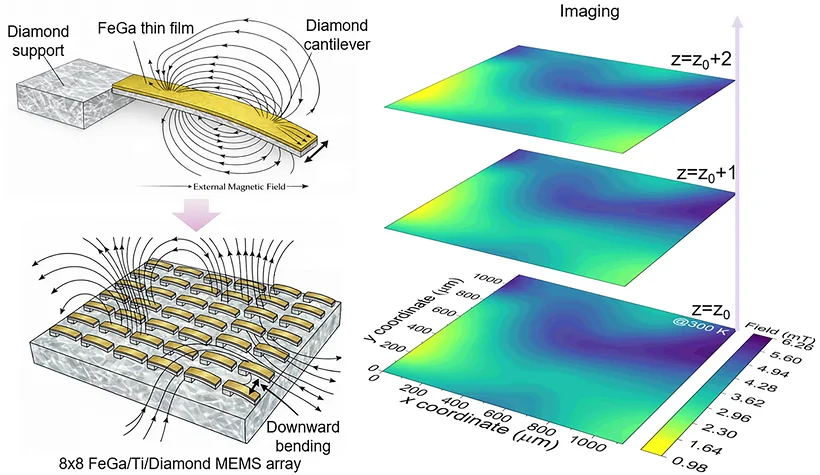

## 1 Introduction

High sensitivity, rapid response, and high spatial resolution are essential for magnetic imaging sensors in detecting magnetic fields, with applications spanning biomedical diagnostics for real‐time neural and cardiac signal detection, geophysical exploration for mapping geomagnetic variations in resource discovery and earthquake prediction, and security screening for non‐invasive detection of concealed magnetic materials [1, 2, 3, 4]. However, achieving precise imaging of ultra‐weak fields in the nanotesla (nT) to femtotesla (*f*T) range remains a challenge, requiring advancements in both sensing materials and device architectures. This challenge becomes particularly intense under harsh high‐temperature conditions encountered in applications such as mineral and oil exploration, space exploration, high‐temperature magnetic valve systems, and automotive engine and transmission speed control systems [5, 6]. Traditional sensors exhibit inherent limitations when applied to high‐resolution magnetic imaging [7, 8]. Fluxgate magnetometers, despite their high sensitivity, suffer from limited spatial resolution due to their large sensor size, making them unsuitable for high‐spatial resolution magnetic field mapping [9, 10]. Magnetoresistive sensors offer better miniaturization but experience severe sensitivity degradation in ultra‐weak fields due to 1/*f* noise and temperature fluctuations, reducing imaging accuracy [11, 12]. Hall sensors, while compact and capable of direct field detection, face a trade‐off between sensitivity and resolution, as material constraints hinder their ability to resolve low‐field variations with high precision [13, 14]. At the high‐sensitivity frontier, Superconducting Quantum Interference Devices (SQUIDs) achieve *f*T‐level detection, making them the benchmark for biomagnetic imaging, yet their reliance on cryogenic cooling results in high power consumption, operational complexity, and susceptibility to electromagnetic interference (EMI), limiting their practicality in unshielded environments [15, 16, 17]. Another promising candidate for high‐performance magnetic imaging employs the nitrogen‐vacancy (NV) center in diamond, a system that exhibits exceptional magnetic sensitivity and nanoscale spatial resolution under ideal conditions. However, practical implementation faces challenges, including the precise control of NV center spin states and the development of robust electrical readout mechanisms for NV‐based signals [18, 19, 20]. These limitations highlight the need for alternative sensing platforms that simultaneously achieve high sensitivity, rapid response, and high‐resolution magnetic imaging while ensuring compact integration, low power consumption, high stability, and practical usability across diverse applications.

Microelectromechanical system (MEMS) resonant magnetic sensors have recently attracted significant attention as a promising alternative to conventional magnetic imaging technologies [21, 22, 23]. These devices offer several intrinsic advantages, including high sensitivity, rapid response, and large throughput, while maintaining compact size and low power consumption. Such characteristics make MEMS‐based approaches particularly appealing for high‐resolution and real‐time magnetic imaging applications. However, the performance of MEMS sensors is strongly influenced by the choice of structural materials. Conventional materials such as silicon (Si) and silicon carbide (SiC), though widely used in MEMS fabrication due to their mature processing technologies, suffer from inherent limitations [24]. Specifically, their brittleness leads to mechanical fragility under high stress or vibration, and their surfaces are difficult to functionalize for specific sensing applications. These drawbacks restrict their integration in advanced imaging systems that demand both high mechanical robustness and versatile surface chemistry. Among emerging MEMS materials, single‐crystal diamond (SCD) stands out as an ideal platform due to its exceptional mechanical properties, including high Young's modulus for enhanced frequency stability, superior thermal conductivity for stable operation, and outstanding chemical inertness for long‐term reliability [25, 26, 27, 28, 29, 30, 31, 32, 33, 34, 35, 36, 37]. Additionally, its intrinsic high resonance frequency operation capability and low energy dissipation with high quality (*Q*) factor allows for the realization of MEMS resonators with high sensitivity and fast response times, both critical for high‐performance weak‐field detection [38]. Furthermore, the low temperature coefficient of frequency (TCF) of SCD resonator is beneficial for suppressing temperature‐induced resonance‐frequency drift and maintaining frequency stability under thermal fluctuations, which is particularly important for resonant sensing at elevated temperatures.

In this work, we present an 8 × 8 magnetic imaging sensor array using FeGa/Ti/SCD cantilever micro‐resonators. By leveraging the Δ*E* effect, where the Young's modulus of FeGa varies under an applied magnetic field, the resonance frequency of the MEMS device undergoes real‐time modulation, providing a highly sensitive, rapid‐response mechanism for weak magnetic field detection. This sensor array exhibits a millisecond‐scale single‐pixel response time of 3.5 ms and magnetic noise level as low as 0.43 nT Hz^−1/2^ at 300 K and 5.45 nT/Hz^−1/2^ at 573 K, respectively. Additionally, the magnetic sensor maintains a minimal frequency fluctuation of ppb level. We demonstrate 2D and 3D magnetic field imaging at temperatures as high as 573 K with a spatial sampling interval of 160 µm using this sensor array. These results demonstrate the potential of SCD MEMS resonators for spatial magnetic‐field reconstruction and high temperature magnetic sensing. This work provides a proof‐of‐concept SCD MEMS resonator array platform for spatial magnetic field sensing under high temperature conditions. The demonstrated platform may provide a basis for future miniaturized magnetic sensing systems intended for high temperature environments.

## 2 Results and Discussion

### 2.1 Device Concept and Architecture

**FIGURE 1:**
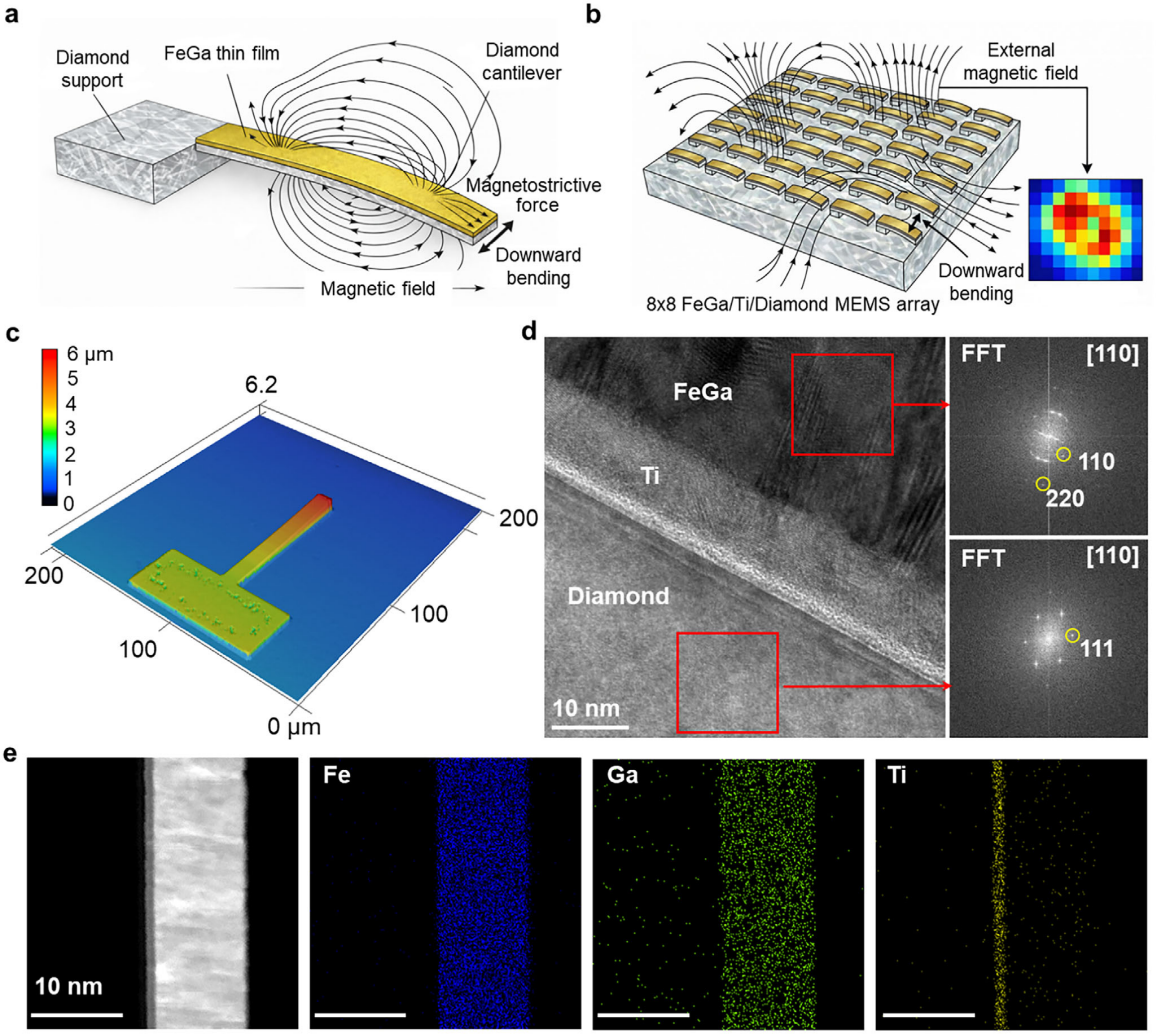
Device structures and sensing principle of SCD‐based resonators. (a) Schematic illustration of a FeGa/Ti/SCD magnetic sensor resonator under an applying magnetic field. The magnetic sensing principle is the magnetostrictive effect (Δ*E* effect). The magnetic response is characterized by the resonance shift. (b) Schematic image of an 8 × 8 SCD‐based magnetic imaging sensor array for magnetic imaging output. Each magnetic sensor within the array function acts as an independent pixel. (c) 3D laser profile of a SCD‐based magnetic sensor resonator. (d) HRTEM image and corresponding fast Fourier transform (FFT) patterns of FeGa/Ti/SCD multilayer structure. (e) EDS mapping of element distributions of this multilayer structure.

The magnetic imaging sensor consists of an array of SCD cantilevers coated with a magnetostrictive FeGa thin film. Each sensor resonator adopts a FeGa/Ti/SCD multilayer configuration (Figure 1a and Figure S1a). The cantilever exhibits a mechanical response to external magnetic fields. By leveraging the Δ*E* effect, wherein the Young's modulus of FeGa is modulated in response to an external magnetic field, the resonance frequency of the SCD cantilever resonators can be finely tuned (Figure S1b). The SCD cantilevers, featuring a monolithic SCD‐on‐SCD architecture, provide a mechanically robust and thermally stable platform for the development of MEMS‐based magnetic transducers. The fabrication process for the SCD resonator‐based magnetic transducer employs a smart‐cut approach combined with ion implantation‐assisted lift‐off technology (Figure S2) [39]. To enable magnetostrictive transduction within the resonator system, a bilayer heterostructure comprising a 10 nm thick titanium (Ti) adhesion layer and a 90 nm thick iron‐gallium (FeGa) magnetic layer was deposited onto bare SCD resonators using magnetron sputtering. The resulting 8 × 8 array of FeGa/Ti/SCD MEMS resonators forms the functional core of the magnetic imaging sensor (Figure 1b). Each magnetic sensor within the array function acts as an independent pixel. The array architecture provides spatially distributed sensing pixels for magnetic‐field reconstruction. The out‐of‐plane profile of the SCD‐based magnetic sensor resonator was characterized by three‐dimensional laser profilometry. The measurement reveals a slight upward curvature of the SCD resonator induced by the release of the sacrificial layer (Figure 1c). High‐resolution transmission electron microscopy (HRTEM) analysis reveals a well‐defined multilayer structure in the FeGa/Ti/SCD stack (Figure 1d), including clean interfaces and an interfacial disordered layer of approximately 0.5 nm between the Ti layer and the diamond substrate. Fast fourier transform (FFT) analysis confirms the (220) crystallographic orientation of the FeGa layer and the (111) facets characteristic of SCD [40, 41]. Elemental mapping further demonstrates the uniform distribution of Fe, Ga, Ti, and C, indicating chemical homogeneity across the multilayer (Figure 1d). The highly oriented FeGa layer is expected to enhance soft magnetic properties, which are crucial for improving sensitivity and performance of SCD‐based resonant magnetic sensing.

**FIGURE 2:**
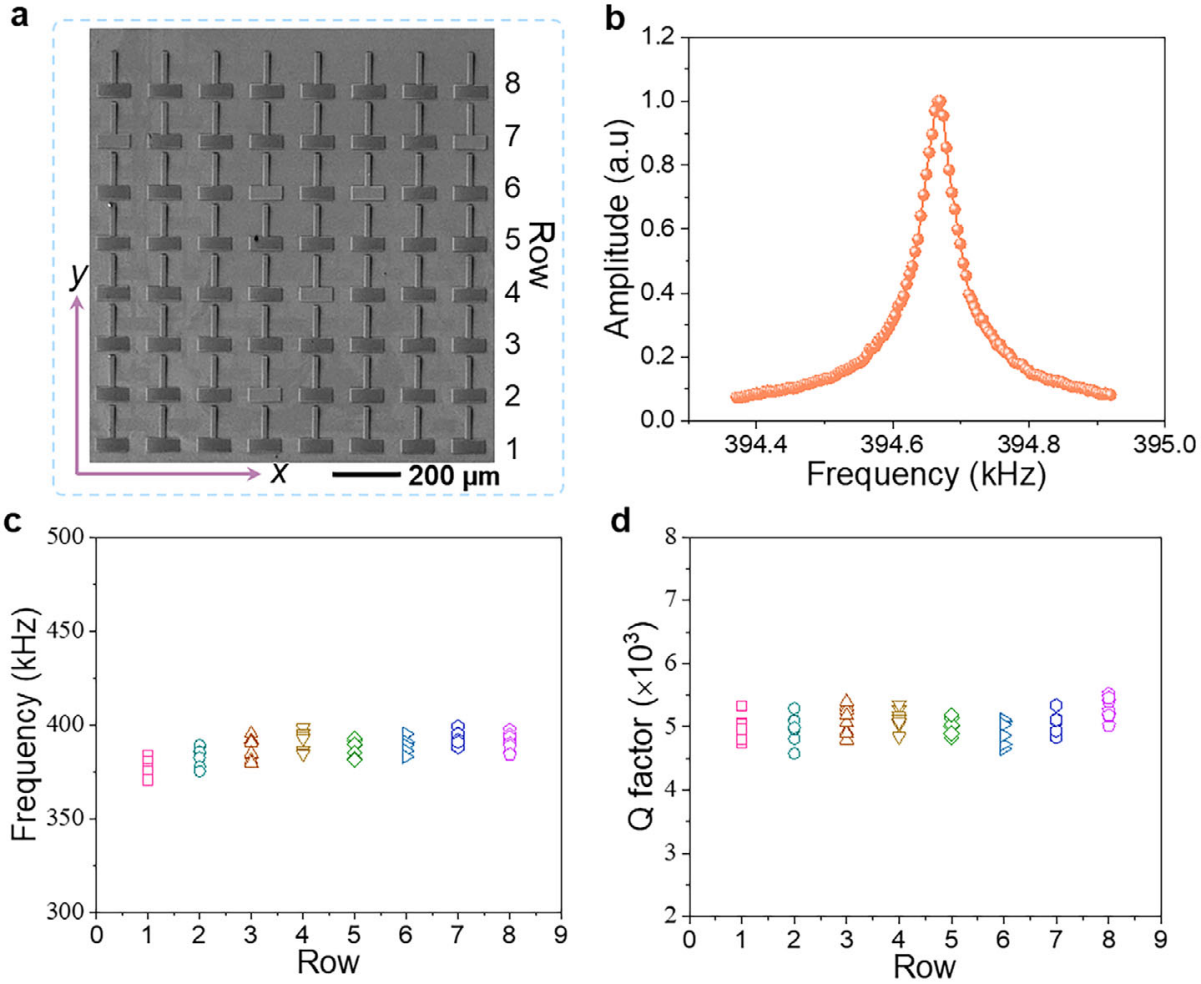
Resonance vibration performance of the 8 × 8 magnetic imaging sensor array. (a) SEM image of this magnetic imaging sensor array. Each sensor row is numbered sequentially. (b) A typical resonance frequency spectrum of an SCD‐based magnetic sensors. (c, d) Resonance frequencies and *Q* factors of magnetic sensors from each row.

The structural configuration of the 8 × 8 SCD cantilever‐type magnetic sensor array is verified by the SEM image shown in Figure 2a. Each resonator unit within the array functions as an independent magnetic field sensor. Each magnetic sensor is assembled as a coordinate (*i*, *j*) in the *x‐y* plane (Figure S3). Before applying the magnetic source, the baseline resonance frequency of each resonator was measured and used as a reference. The magnetic‐field‐induced resonance‐frequency shift was subsequently determined relative to this initial state. The resonance frequency shift of the sensor unit with the coordinate (*i*, *j*), Δ*f*_ij_, generated by applying a magnetic field can be expressed as,

(1) $$\Delta f_{ij}=\left|f_{ij}^{H}-f_{ij}^{0}\left|=\right|\frac{K_{n}^{2}}{4\pi \sqrt{3}}\frac{t}{L^{2}\sqrt{\rho }}\left(\sqrt{E_{ij}^{H}}-\sqrt{E_{ij}^{0}}\right)\right|$$

wherein *t* and *L* are the thickness and effective length of the resonator sensor. *E*_H_ and *E*_0_ are the Young's modulus of the sensor with and without a magnetic field, respectively. The first vibration mode of the resonator was employed with *K*_1_ = 1.8751 [27, 42]. *f*_ij, H_ and *f*_ij, 0_ represent the resonance frequencies of sensor unit (*i*, *j*) with and without a magnetic field. The magnetic sensitivity (𝑆_ij_) of each resonator sensor is quantified as the resonance frequency shift per unit magnetic field, Δ*H*_ij_,

(2) $$S_{ij}=\frac{\Delta f_{ij}}{\Delta H_{ij}}$$

Using the resulting sensitivity and frequency shift, the measured magnetic field for each sensor can be identified. The magnetic fields corresponding to each sensor located at coordinate (*i*, *j*) were employed to construct the magnetic field image. Before magnetic‐field reconstruction, each resonator pixel was individually calibrated under a known external magnetic field to determine its magnetic sensitivity *S*_ij_. The measured resonance frequency shift Δ*f*_ij_ was then converted into the local magnetic field *H*_ij_ according to: *H*_ij_ = Δ*f*_ij_/*S*_ij_. This pixel‐by‐pixel calibration compensates for the sensitivity variation among resonators and improves the consistency of the reconstructed magnetic‐field maps. In addition, the uncertainty in the reconstructed magnetic‐field values mainly originates from sensor‐to‐sensor sensitivity variation, resonance frequency fitting uncertainty, and baseline drift during sequential optical acquisition. We note that the present magnetic‐field maps are a proof‐of‐concept demonstration rather than fully optimized quantitative magnetic images. Further improvements in quantitative imaging reliability will require repeatability analysis, drift correction, and scalable parallel readout architectures. Thermal drift effects during long acquisition processes were not separately quantified in the present work and will be investigated in future work.

### 2.2 Resonance Characteristics of Diamond‐Based Magnetic Sensor Array

The micromechanical resonance characteristics of the magnetic sensor array were evaluated using laser Doppler vibrometry (Figure S4). A piezoelectric (PZT) actuator was employed to enable multi‐point magnetic field mapping by actuating the cantilever resonators. To facilitate magnetic sensing, external magnetic fields were applied using a coil pair driven by AC/DC power sources. This experimental configuration allows the resonance response of individual sensing pixels to be measured sequentially for spatial magnetic‐field reconstruction, enabling magnetic imaging of complex magnetic field distributions. The resonators exhibited a linear amplitude response to the applied RF excitation, while their resonance frequencies remained stable throughout the tests, confirming the elastic linearity of the system (Figure S5). Despite the deposition of FeGa/Ti layers, the *Q* factors of the diamond resonators remained high, ranging from approximately 4000 to 6000, indicating minimal damping and strong mechanical performance suitable for high‐sensitivity magnetic detection.

The magnetic sensor rows within the array are arranged sequentially to enhance spatial clarity during data interpretation (Figure 2a). A typical resonance spectrum obtained from an SCD‐based magnetic sensor exhibit sharp, well‐defined resonance peaks (Figure 2b). The variation in resonance frequency from sensor‐to‐sensor is around 5% (Figure 2c), reflecting the high uniformity of the sensor array. Moreover, the *Q* factor of each magnetic sensor remains highly consistent (Figure 2d). The observed fluctuation in *Q* values ranging from approximately 4500 to 5500 underscores the consistent mechanical performance of the SCD‐based resonator array. These results demonstrate relatively uniform resonance characteristics and consistent mechanical performance across the fabricated 8 × 8 sensor array.

### 2.3 Magnetic Response of Magnetic Imaging Sensor Array

**FIGURE 3:**
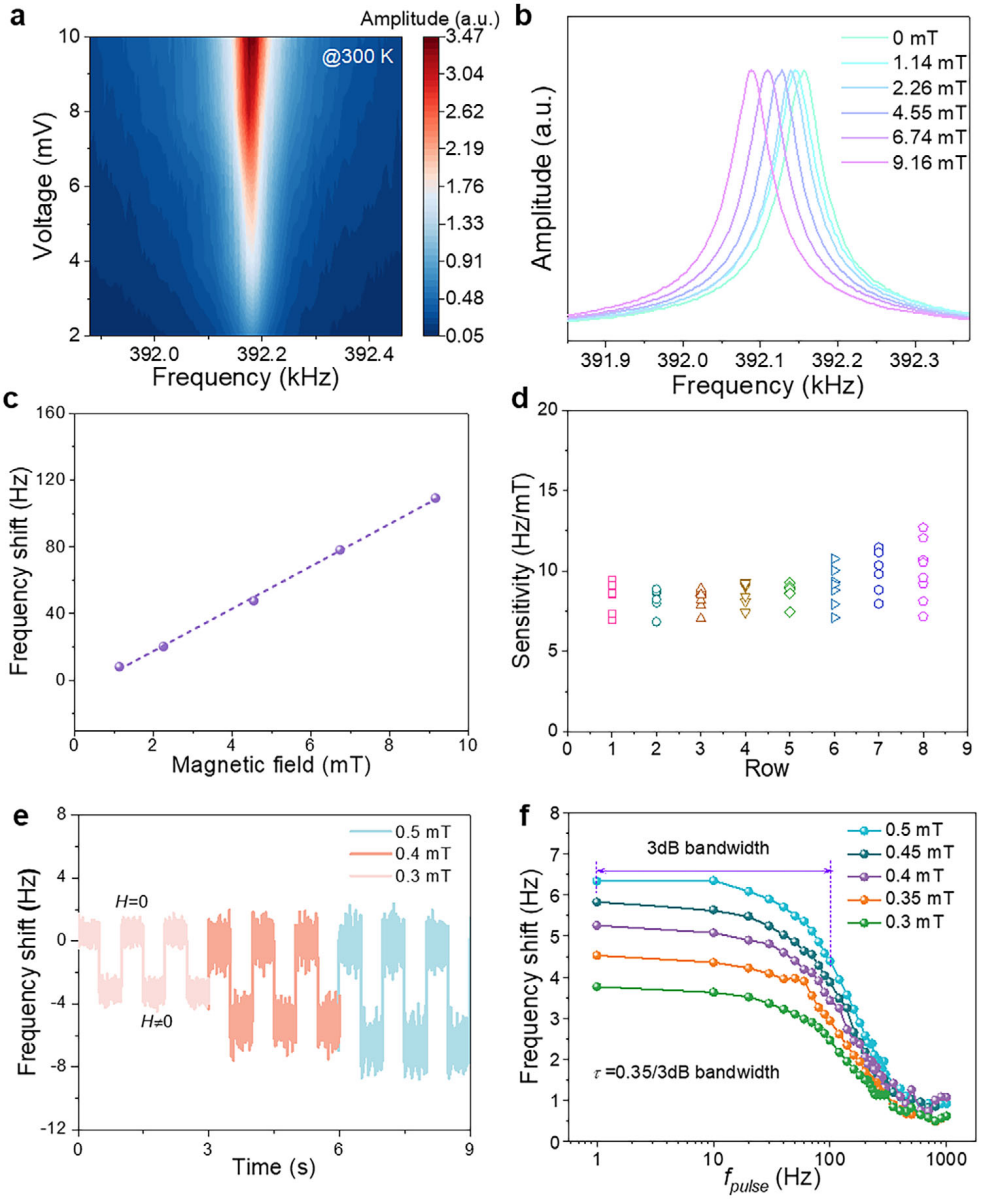
Magnetic sensing performance of the 8 × 8 magnetic imaging sensor array under room temperature. (a) Dependence of resonance frequency spectra of a 100 µm‐long SCD resonator on actuation voltages. The color indicates the resonance amplitude. (b) Variation Resonance spectrum of a SCD‐based magnetic sensor as a function of magnetic field. (c) Dependences of resonance frequency shifts on magnetic field. The slope of the linear fitting line is used to indicate the magnetic sensitivity of each magnetic sensor. (d) Magnetic sensitivities of the sensor within each row. (e) Time‐dependent magnetic‐response of a SCD‐based magnetic resonator sensor under various magnetic fields. The pulse frequency of applying the magnetic field is 2 Hz. (f) Dependences of resonance frequency shift of a SCD‐based magnetic sensor on the pulse frequency of applying magnetic field under various magnetic fields.

Each magnetic sensor in the array responds to external magnetic fields through the magnetostrictive effect. The magnetic sensitivity is defined as the frequency shift vs magnetic field, as expressed in Equation (2). The actuation voltage has negligible impact on the resonance frequency, resulting in a linear increase in vibration amplitude (Figure 3a). As the magnetic field strength increases, the resonance spectrum exhibits a downshift toward lower frequencies at 300 K (Figure 3b and Movie S1), indicating a field‐dependent frequency response. Individual sensors exhibit linear frequency shifts, with a sensitivity of 12.7 Hz/mT at 300 K (Figure 3c). Consistent performance is observed across the sensor array (Figure S6), with sensitivities ranging from 7.2 to 12.7 Hz/mT (Figure 3d). Dynamic performance was evaluated by applying pulsed magnetic fields, showing that the sensor demonstrates a rapid magnetic response (Figure 3e). To assess the high‐speed sensing capability, magnetic fields with various frequencies were applied to the magnetic sensor. The sensor's response time was determined using the 3 dB method, where the 3 dB bandwidth is defined as the frequency, *f*_3dB_ at which the resonance amplitude decreases to 70.7% of its reference value [43, 44]. The response time, *τ* is inversely related to the 3 dB bandwidth and is expressed as: *τ* ≅ 0.35/*f*_3dB_. At a magnetic field strength of 0.5 mT, the sensor exhibits a 3 dB bandwidth of 100 Hz (Figure 3f), corresponding to a rapid response time of 3.5 ms. The response time is similar to the decay time of the SCD resonator. Furthermore, we examined the influence of different magnetic field strengths on the sensor's response time and confirmed its consistent high‐speed performance across a range of fields (Figure S7). The rapid response of the magnetic sensor can be attributed to three primary factors: (1) high magnetostrictive coefficient and low magnetic hysteresis of the FeGa film, allowing fast magnetization dynamics under applied fields; (2) exceptional mechanical stability of the SCD micromechanical resonator, enabling low‐noise, high‐frequency operation ideal for rapid magnetic detection; and (3) efficient magnetoelastic coupling through the Ti interlayer, which serves as both a mechanical buffer and adhesion promoter, enhancing sensitivity and response speed. Collectively, the FeGa/Ti/SCD resonator architecture provides a resonant platform for rapid magnetic‐field sensing.

**FIGURE 4:**
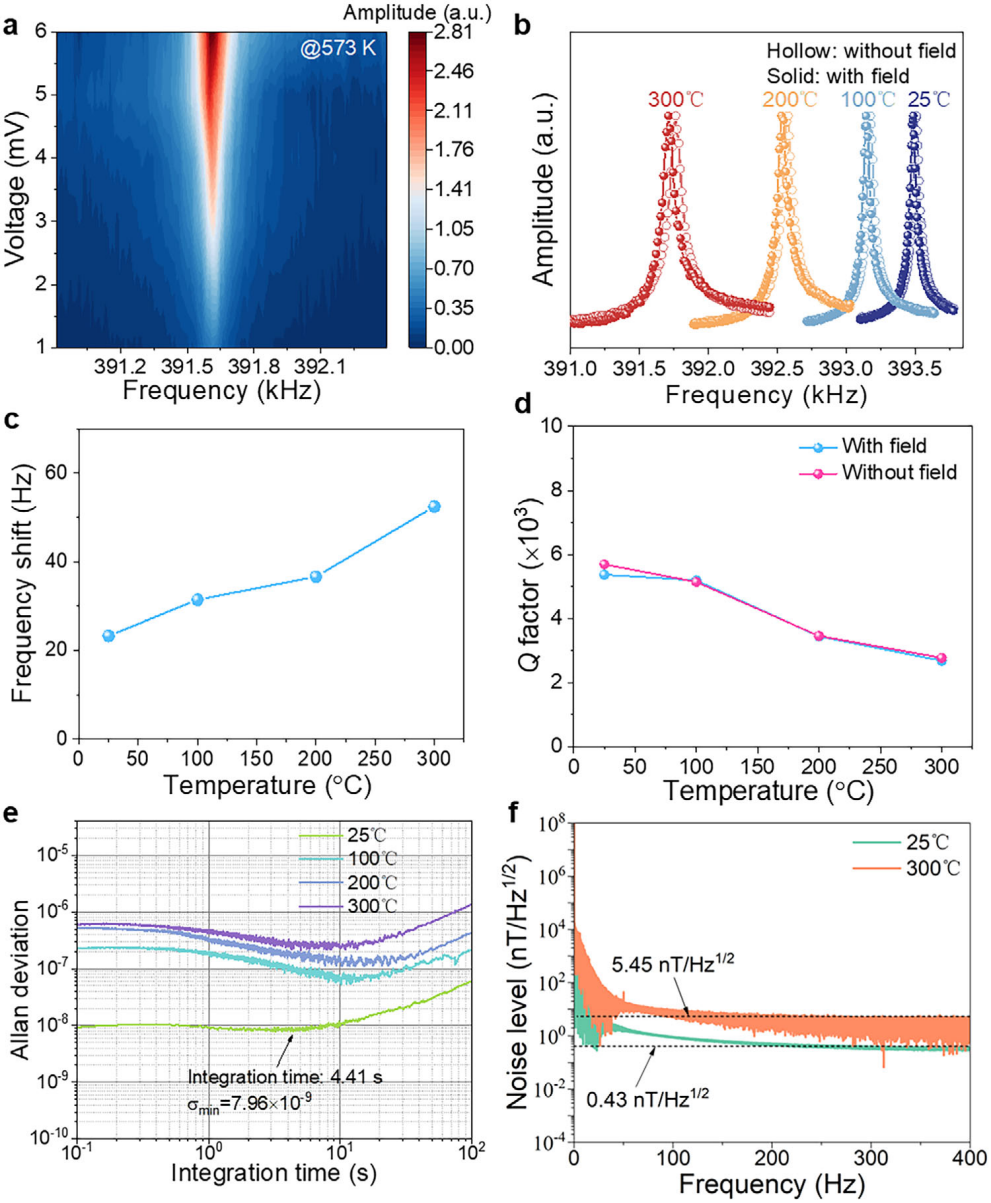
High‐temperature magnetic sensing performance of the SCD‐based magnetic sensor. (a) Resonance frequency spectrum of a 100 µm‐long SCD‐based magnetic sensor as a function of actuation voltage at 573 K. (b) Resonance frequency shifts of the sensor measured across different temperatures. (c) Temperature dependence of the resonance frequency shifts. (d) *Q* factor variation of the SCD‐based magnetic sensor as a function of temperature. (e) Dependences of the Allan deviation of an SCD‐based magnetic sensor on measurement temperature. (f) Magnetic noise spectra measured at 300 K and 573 K with the target bandwidth of 1 Hz.

We investigated the magnetic sensing performance of FeGa/Ti/SCD‐based sensors at high temperatures, utilizing the magnetostrictive effect as the transduction mechanism. The resonance frequency spectrum measured at 573 K reveals a linear increase in peak amplitude with increasing actuation voltage, consistent with the behavior observed at 300 K (Figure 4a). This consistency indicates stable actuation dynamics across the investigated temperature range. The influence of external magnetic fields on the resonance characteristics at elevated temperatures is presented (Figure 4b). The resonance spectrum shifts toward lower frequencies upon applying a magnetic field and returns to its original peak position upon removal of the magnetic field (Movies S2 and S3). The magnetic‐field‐induced resonance frequency shift, as defined in Equation (1), demonstrates a slight enhancement with increasing temperature (Figure 4c), reaching a maximum sensitivity of 28.58 Hz/mT at 573  K. Notably, the *Q* factor remains largely unaffected by the applied magnetic field (Figure 4d). However, a temperature‐ dependent decrease in the *Q* factor is observed, which is attributed to elevated intrinsic damping mechanisms at higher temperatures. In sensing applications, the smallest detectable frequency shift, Δ*f*_min_, is limited by the noise [45]. Allan deviation is employed as a statistical tool to evaluate the frequency stability of the SCD‐based magnetic sensor over varying averaging times. The Allan deviation is defined as the square root of the Allan variance, which is expressed as [45, 46],

(3) $$\sigma_{A}\;\left(\tau \right)=\sqrt{\frac{1}{2\left(N-1\right)}{\sum_{i=1}^{N-1}\left(\frac{\bar{f_{i+1}}-\bar{f_{i}}}{f_{0}}\right)}^{2}}$$

where *N* represents the total number of resonance frequency samples, *f*_1_, *f*_2_,..., *f*_N_, each calculated as an average over the integration time *τ*. The Allan deviations of an SCD‐based magnetic sensor at evaluated temperatures are presented in Figure 4e. The analysis reveals that the minimum frequency fluctuation reaches as low as 7.96×10^−9^ and 2.13×10^−7^ at 300 K and 573 K, respectively, which is used to estimate the minimum detectable magnetic field. Based on the experimentally measured magnetic sensitivity and this frequency stability, the minimum detectable magnetic fields are determined to be 244.3 nT and 2919.3 nT at average times of 4.41 s and 5.04 s under 300 K and 573 K, respectively. Further evaluation of the sensor's magnetic noise characteristics was performed at both 300 K and 573 K. The magnetic noise spectra of a 100 µm‐long FeGa/Ti/SCD resonator reveal a low noise floor of 0.43 nT Hz^−1/2^ at 300 K and 5.45 nT/Hz^−1/2^ at 573 K, respectively (Figure 4f). The time‐dependent resonance frequencies of the magnetic sensor cantilever at 300 K and 573 K are shown in Figure S8. It should be noted that the spectral magnetic noise density and the Allan‐deviation‐derived minimum detectable magnetic field describe different aspects of sensor performance. The spectral magnetic noise density, expressed in nT Hz^−1/2^, characterizes the short‐term frequency‐domain noise performance normalized to a 1 Hz bandwidth. By contrast, the minimum detectable magnetic field estimated from Allan deviation analysis reflects the quasi‐static or DC magnetic‐field resolution at a specific averaging time and is strongly influenced by low‐frequency drift, temperature fluctuation, and long‐term frequency stability. Therefore, these two quantities should not be treated as interchangeable metrics. The temperature‐dependent resonance behavior of the FeGa/Ti/SCD resonator also reflects the low TCF of the FeGa/Ti/SCD platform (Figure S9). A low TCF is beneficial for reducing thermally induced resonance‐frequency drift and improving frequency stability under elevated‐temperature operation, which is particularly important for resonant magnetic sensing applications. Previous work on individual SCD resonators has shown stable resonance behavior under repeated heating/cooling cycles [47]. Although such measurements were not performed on the present array structure, these resulted support the intrinsic thermal stability of SCD MEMS resonators. While magnetic sensing and spatial field mapping were demonstrated up to 573 K, a systematic evaluation of array‐level thermal robustness remains necessary. Future works should include repeated thermal cycling, long‐term drift analysis, and denser temperature‐dependent measurements. The advantage of the SCD platform is not primarily reflected in achieving the highest room‐temperature sensitivity or the lowest reported detection limit (Table S1). Rather, its significance lies in enabling resonant magnetic sensing under elevated‐temperature conditions, where resonance stability becomes increasingly important. The ability to maintain magnetic sensing functionality up to 573 K distinguishes the present platform from most previously reported Δ*E*‐effect MEMS magnetic sensors.

### 2.4 Magnetic Imaging

To enable two‐ and three‐dimensional magnetic field imaging under dynamic thermal conditions, an 8 × 8 magnetic sensor array based on FeGa/Ti/SCD cantilevers was developed. Each sensor within the array function acted as an independent pixel and consists of an SCD MEMS resonator integrated with a FeGa/Ti bilayer. In the present proof‐of‐concept experiment, the array was read out sequentially using laser Doppler vibrometry. Therefore, the reconstructed magnetic images represented spatial field maps obtained from calibrated individual pixels, rather than full‐frame images acquired by simultaneous parallel readout. The current concept of the array‐based sensing architecture for spatial magnetic‐field reconstruction provides a base for a fully integrated imaging system with parallel array readout. The reported response time of 3.5 ms corresponds to the intrinsic response of an individual resonator pixel under pulsed magnetic excitation. Future development of the SCD‐based magnetic sensor array toward real‐time full‐frame magnetic imaging will require scalable parallel or multiplexed electrical readout architectures [48].

**FIGURE 5:**
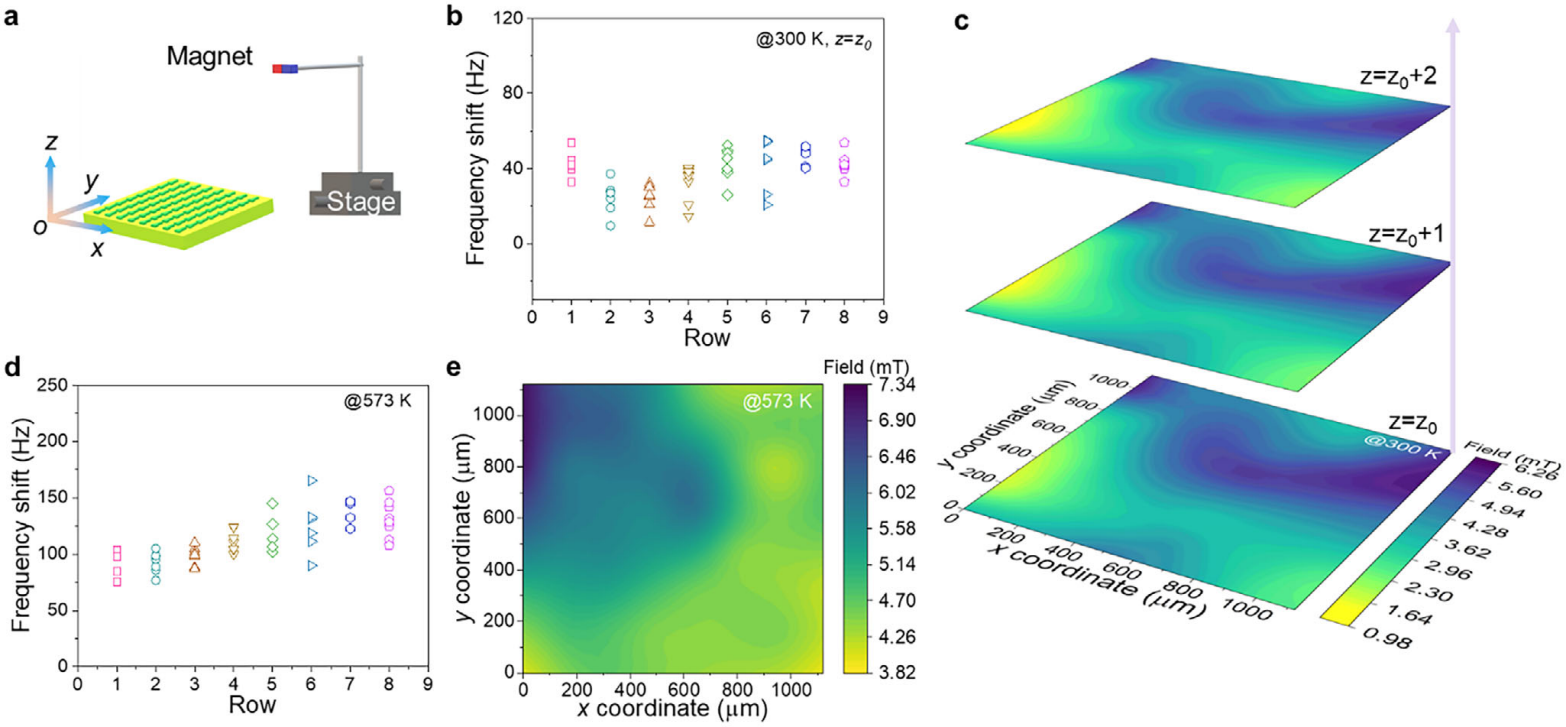
(a) 3D magnetic field mapping, where the magnet is moved along the *z* coordinate at 1 mm interval. The magnet is initially located *z*_0_ = 10 mm above the horizontal surface of the magnetic sensor array. (b) Resonance frequency shifts of SCD‐based magnetic sensors within each row as *z =* *z*_0_ at 300 K. (c) 3D Magnetic field imaging of a magnet via the magnetic sensor array with changing position of the magnet along *z* coordinate as *z*_0_, *z*_0_+1 mm, and *z*_0_+2 mm. The evaluation temperature is 300 K. (d) Resonance frequency shifts of SCD‐based magnetic sensors within each row at 573 K. (e) Magnetic imaging of the SCD‐based magnetic sensor array at 573 K.

Using the calibrated magnetic sensitivity of each sensor, the measured frequency shifts were converted into spatial magnetic‐field distributions. Under the present sequential optical readout scheme, acquisition of the magnetic sensor array dataset required approximately 6.6 s, while subsequent field‐map reconstruction and visualization required approximately 1 min. In the future, the magnetic mapping duration could be markedly reduced by using electrical readout. The magnetic field source, a 3  ×  3  ×  8  mm^3^ (length × width × height) shaped magnet, is translated along the *z*‐axis using a motorized 3D stage in 1 mm increments, with the initial separation from the array set at *z*_0_ =  10 mm (Figure 5a). The resulting resonance frequency shifts of the sensor array at *z* = *z*_0_ and 300 K are shown in Figure 5b. Additional data for *z* = *z*_0_+1 mm and *z*_0_+2 mm are provided in Figure S10 and Movie S4. Using the known sensitivity of each sensing unit, the local magnetic field *H*_(i,j)_ at position (*i,j*) is calculated from the measured frequency shift Δ*f*_(i,j)_. Figure 5c presents the reconstructed 3D magnetic field distributions over a 1120 × 1120 µm^2^ sensing area, recorded at three vertical positions of the magnet (*z*_0_, *z*_0_+1 mm, and *z*_0_+2 mm). The multiple *z* positions were applied to demonstrate magnetic field mapping along the vertical direction. As the magnet‐to‐sensor distance increases, the reconstructed magnetic‐field intensity decreases, confirming that the array can capture the spatial decay of the magnetic field and enabling proof‐of‐concept three‐dimensional magnetic field mapping. The current magnetic‐field reconstruction is based on an array with a center‐to‐center pixel spacing of 160 µm. Therefore, the demonstrated spatial mapping capability is limited by the sampling interval of the array. This pitch‐limited sampling interval should not be interpreted as the intrinsic spatial‐resolution limit of the sensing mechanism. In addition, the measured field distribution is influenced by the geometry of the magnetic source and the source‐to‐sensor separation. Because the permanent magnet used in the present work has millimeter‐scale dimensions and is positioned several millimeters above the array, the measured magnetic‐field distribution is spatially broadened.

Future implementation of full‐frame magnetic imaging will require scalable array‐level interfacing and readout architectures. Possible directions include multiplexed electrical signal acquisition and integrated on‐chip transduction structures. In addition, pixel addressing, crosstalk suppression, and throughput optimization need to be considered for practical system development. To examine the consistency of magnetic‐field reconstruction under a controlled change in source orientation, the same 3 × 3 × 8 mm^3^ permanent magnet was rotated by 90° in the plane parallel to the sensor array, corresponding to rotation about the *z*‐axis normal to the array plane. The magnetization direction, *M*, which is along the longitudinal axis of the magnet, rotated together with the magnet. The magnet center and source‐to‐sensor distance were maintained during rotation. A corresponding change in the reconstructed spatial magnetic‐field distribution is observed in Figure S11, indicating that the array can capture changes in the field distribution associated with the orientation of the magnetic source. In addition, magnetic‐field distributions were measured at different *z* positions. Rotation of the magnetic source resulted in a corresponding change in the spatial field distribution, while increasing the *z* distance led to a reduction and spatial broadening of the magnetic‐field intensity. These observations indicate that the sensor array can capture changes in spatial magnetic‐field distributions. Owing to the high thermal stability of the FeGa/Ti/SCD multilayer structure, the sensor array can also operate at high temperatures. The magnetic sensitivities of the magnetic sensor array measured at 573 K are provided in Figure S12. These temperature‐matched calibration values were used to reconstruct the magnetic‐field distribution at 573 K, thereby accounting for both sensor‐to‐sensor variation and the temperature dependence of magnetic sensitivity. This capability is demonstrated by successful magnetic field imaging of a fixed‐position magnet at 573 K (Figure 5d,e), clearly showing high and low magnetic fields intensity contrast. These results demonstrate magnetic‐field sensing and spatial field reconstruction at 573 K, supporting the elevated‐temperature operability of the SCD MEMS resonator array. Finally, Table 1 compares the performance characteristics of the present sensors array with representative magnetic imaging technologies. The comparison illustrates the combination of millisecond single‐pixel response, nanotesla‐level magnetic noise, and operation up to 573 K demonstrated by the present sensor array, making it a candidate for next‐generation magnetic imaging applications in extreme environments. Several factors may affect the quantitative accuracy of magnetic‐field reconstruction, including alignment uncertainty, stage positioning accuracy, and thermal drift during sequential measurements. Although these effects were not independently quantified in the present work, they should be considered in future optimization of the imaging platform.

**TABLE 1:** Comparison of various magnetic field imaging sensors.

Magnetic imaging sensor	Sensitivity	Spatial resolution/sampling interval	Response time	Working temperature	Refs.
Optically‐pumped magnetometer	24 fT/Hz^1/2^	1.5 mm	NA	25°C	[49]
Scanning NV centre microscopy	3 µT/Hz^1/2^	6 nm	0.6 s/7.5 s	<‐267°C	[50]
Scanning SQUID microscopy	130 nT/Hz^1/2^	0.5 µm	NA	<‐264°C	[51]
Magnetic force microscopy	170 nT/Hz^1/2^	10–100 nm	NA	<227°C	[2]
Hall‐bar microscopy	500 µT/Hz^1/2^	100 nm	100 ms	−264°C	[52]
MEMS microscopy	5.45 nT/Hz^1/2^	160 µm (sampling interval)	3.5 ms	300°C	This work

## 3 Conclusions

In summary, we report an 8 × 8 magnetic‐field sensing array based on SCD resonators, with magnetic sensing operation up to 573 K. The sensor array exhibits a single‐pixel response time of 3.5 ms, magnetic noise levels of 0.43 nT Hz^−1/2^ at 300 K and 5.45 nT Hz^−1/2^ at 573 K, and frequency stability at the parts‐per‐billion level. Two‐ and three‐dimensional magnetic‐field reconstruction is demonstrated with a spatial sampling interval of 160 µm. These results demonstrate the feasibility of using SCD MEMS resonator array for spatial magnetic‐field reconstruction and elevated‐temperature magnetic sensing, and provide a basis for the further development of miniaturized magnetic‐field sensing systems for elevated‐temperature environments.

## 4 Experimental Section

### 4.1 Devices Fabrication

In this work, the smart‐cut technique was employed to fabricate the SCD resonators, following technologies outlined in previous works [53, 54]. The process began with the growth of a diamond epilayer on high‐pressure high‐temperature (HPHT) SCD substrates. Before diamond growth, the HPHT SCD substrates underwent a thorough cleaning process involving sequential boiling in acid mixtures (H_2_SO_4_+HNO_3_), followed by rinses in acetone, ethanol, and deionized water to remove surface contaminants. Subsequently, carbon ions were implanted into the cleaned SCD substrates at an energy of 180 keV and a dose of 10^16^ cm^−2^. Following the implantation, diamond epilayers were grown on the substrates using a microwave chemical vapor deposition (MPCVD) system. The growth parameters were as follows: 500 sccm hydrogen flow rate, 0.5% methane concentration in hydrogen, 1000 W microwave power, a working temperature of 840°C, and a growth time of 3 h. During the diamond growth, ion implantation induced the formation of a ∼200 nm thick graphite‐like layer beneath the diamond surface. This layer functioned as a sacrificial layer to release the resonator structure. The presence and morphology of this layer were confirmed using transmission electron microscopy (TEM), as reported in our earlier studies [53, 54]. Then, a 150 nm‐thick aluminum (Al) film was deposited onto the SCD epilayer to serve as a metal mask, patterned using laser lithography [27]. The patterned diamond was then dry‐etched using an inductively coupled plasma (ICP) reactive ion etching system in a pure oxygen environment. Following the etching process, the Al mask was removed using a boiling acid mixture (H_2_SO_4_+HNO_3_), which simultaneously facilitated the release of the suspended resonator structures. To improve the material quality and reduce defects induced by ion implantation, the fabricated SCD samples were annealed at 1100°C for 3 h under ultra‐high vacuum conditions (<10^−7^ Pa). This post‐processing step significantly enhanced the *Q* factors of the SCD resonators. Finally, FeGa and Ti films were sequentially deposited onto the diamond resonators using a radio‐frequency magnetron sputtering system. The sputtering process was conducted under the following conditions: Ar flow rate of 10 sccm, working pressure of 1 Pa, sputtering power of 100 W, and at 300 K. The resulting FeGa/Ti/SCD multilayer resonator architecture was employed for magnetic sensing applications.

### 4.2 Materials and Devices Characterization

The three‐dimensional (3D) surface profiles of the SCD resonators were examined using a 3D laser scanning optical microscope (VK‐9710). Optical spectral measurements were performed with a spectrometer equipped with an 1800 lines/mm monochromator grating and a thermoelectrically cooled charge‐coupled device (CCD) detector. An optical system using the Doppler effect of a focused He‐Ne laser (633 nm, <1 mW) was employed to measure the out‐of‐plane resonance frequencies of the SCD resonators. The lock‐in amplifier system was used to read out the resonance signal. All measurements were conducted in a vacuum chamber with a pressure below 10^−3^ Pa. The sensor array was actuated by a piezoceramic connected to an RF signal, enabling controlled excitation of the resonators across a temperature range from 300 K to 573 K. The temperature of 573 K was selected because it represents a practically important performance for high‐temperature MEMS sensing. Conventional silicon‐based MEMS platforms often encounter significant thermal drift, packaging‐related stress, and reliability issues near or above this temperature range. Magnetic fields for sensitivity calibration were generated by a pair of Cu coils. To assess temporal response characteristics, pulsed current signals were applied via a function generator (IWATSU, SG‐4105). For magnetic field mapping, the permanent magnet (dimensions: 3 × 3 × 8 mm^3^, length × width × height) was mounted on a motorized three‐dimensional translation stage and positioned above the center of the 8 × 8 resonator array. The vertical distance z was defined as the separation between the lower surface of the magnet and the plane of the resonator array (Figure S13). Before field mapping, the baseline resonance frequency of each resonator was recorded without the magnet. The magnet was then positioned at *z*_0_ = 10 mm and translated along the *z* direction with 1 mm step increments. The background frequency shift was subtracted from the measured response to obtain the magnet‐induced resonance‐frequency shift. For the magnetic‐field mapping at 573 K, each resonator pixel was calibrated at 573 K, and the corresponding temperature‐specific sensitivity was used for reconstruction of the local magnetic‐field distribution. The resonator pixels were interrogated sequentially using a focused laser Doppler vibrometer (Polytec MSA‐600) following a row‐by‐row acquisition sequence. Repositioning of the laser spot between adjacent sensors required approximately 100 ms. Considering the single‐pixel magnetic response time of approximately 3.5 ms, acquisition of the magnetic sensor array dataset required approximately 6.6 s under the present measurement configuration. Subsequent reconstruction and visualization of the magnetic‐field distribution were performed using Origin, requiring approximately 1 min. For visualization of the magnetic‐field distribution, the reconstructed magnetic‐field values at the array sensing pixels were interpolated in Origin using the Data Boundary contouring method to generate continuous contour maps. A smoothing parameter of 0.05 and a total points increase factor of 100 were applied only for graphical rendering. The interpolation does not modify the measured pixel values or introduce additional spatial sampling information beyond the original measurement grids. For spectral‐noise characterization, the resonance frequency was continuously tracked using a phase‐locked loop (PLL) for 300 s at a data‐transfer rate of 899.5 Sa s^−1^. The PLL target bandwidth was set to 1 Hz. The recorded resonance‐frequency fluctuations were transformed into the frequency domain using a FFT and normalized to obtain the one‐side frequency‐noise amplitude spectral density 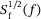. The equivalent magnetic‐noise amplitude spectral density was calculated as 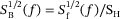, where *S*_H_ is the experimentally measured magnetic sensitivity at the corresponding temperature.

## Author Contributions

**Zilong Zhang**: conceptualization, methodology, data curation, investigation, validation, formal analysis, writing – original draft, writing – review and editing. **Keyun Gu**: writing – review and editing. **Satoshi Koizumi**: writing – review and editing. **Masaya Toda**: writing – review and editing. **Meiyong Liao**: conceptualization, methodology, supervision, writing – original draft, project administration, writing – review and editing, data curation, validation, formal analysis.

## Conflicts of Interest

The authors declare no conflicts of interest.

## Supporting information




**Supporting File 1**: advs77779‐sup‐0001‐SuppMat.docx.

**Supporting File 2**: advs77779‐sup‐0002‐VideoS1.mp4.

**Supporting File 3**: advs77779‐sup‐0003‐VideoS2.mp4.

**Supporting File 4**: advs77779‐sup‐0004‐VideoS3.mp4.

**Supporting File 5**: advs77779‐sup‐0005‐VideoS4.mp4.

## Data Availability

The data that support the findings of this study are available from the corresponding author upon reasonable request.
