# Supplementary material for: High‐Temperature Magnetic Field Mapping Using a Single‐Crystal Diamond MEMS Resonator Array
Source: Adv Sci (Weinh). 2026 Sep 16:e77779. Online ahead of print. doi: 10.1002/advs.77779 (PMC13583230; doi:10.1002/advs.77779)
Supplement: Supplementary file 1 [file ADVS-9999-e77779-s004.docx]

**Supporting Information**

**High-Temperature Magnetic Field Mapping Using a Single-Crystal Diamond MEMS Resonator Array**

Zilong Zhang^1,2^*, Keyun Gu^1^, Satoshi Koizumi^1^, Masaya Toda^3^ and Meiyong Liao^1,4^*

^1^ Research Center for Electronic and Optical Materials, National Institute for Materials Science (NIMS), 1-1 Namiki, Tsukuba, Ibaraki 305-0044, Japan.

^2^ Institute of Metal Research Chinese Academy of Sciences, 72 Wenhua Road, Shenyang 110016, China.

^3^ Graduate School of Engineering, Tohoku University, Sendai, Miyagi 9808579, Japan

^4^ University of Tsukuba, Tsukuba, Ibaraki 305-8571, Japan

Correspondence should be addressed to Zilong Zhang* (Email: [zlzhang@imr.ac.cn](mailto:zlzhang@imr.ac.cn)) and Meiyong Liao* (Email: meiyong.liao@nims.go.jp)

**1. Device structures and magnetostrictive effect (ΔE effect) of multilayer resonators**

**Figure S1. a** Schematic illustration of the magnetic imaging sensors array with a zoomed structure image of a magnetic sensor resonator. **b** Magnetic sensing principle of magnetostrictive effect (ΔE effect). The magnetic response is characterized by the resonance shift.

**2. Fabrication of single-crystal diamond (SCD) resonator**

3

**Figure S2.** Fabrication of diamond resonators. **a** Mix-acid cleaning of high-pressure high-temperature (HPHT) single-crystal diamond (SCD). **b** Ion implantation of SCD substrate. **c** SCD epilayer growth through microwave plasma chemical vapor deposition (MPCVD). **d** Spin-coating of photoresist. **e** Patterning by photolithography. **f** Deposition of metal film. **g** Lift-off of photoresist. **h** Inductively coupled plasma (ICP) reactive ion etching (RIE). **i** Mix-acid etching of graphite and metal film.

**3. Optical image of the magnetic sensors array**

**Figure S3. a** Laser optical image of SCD-based magnetic sensors array. **b** The coordinate of each SCD based magnetic sensor in the *x-y* plane.

**4. Measurement setup of micro-resonator through the Doppler effect**

**Figure S4.** Schematic illustration of the measurement setup of the SCD cantilever with an optical readout system.

**5. Resonance spectra of a bare diamond micro-resonator**

**Figure S5.** **a** Dependence of driven voltage on resonance spectrum of a SCD cantilever without the deposition of FeGa/Ti film. **b** The vibration amplitude as a function of driven voltage. The resonance amplitude increases linearly with the driven voltage.

**6. Resonance frequency shifts of the magnetic sensors array with varying magnetic fields**

**Figure S6.** Dependences of resonance frequency shifts of SCD-based magnetic sensors in each row within the array structure on magnetic field.

**7. Response time of a SCD-based magnetic sensor under different magnetic fields**

**Figure S7.** The variation of response time of the SCD-based magnetic sensor with magnetic fields.

**8. Variation of the resonance frequency of the magnetic sensor cantilever over time at 25 °C and 300 °C**

**Figure S8.** Time-dependence resonance frequency of the cantilever magnetic sensor under 25 ℃ and 300 ℃, respectively.

**9. Temperature coefficient of resonance frequency (TCF) of FeGa/Ti/SCD magnetic sensor**

**Figure S9**. Temperature dependence of resonance frequency and extracted TCF of FeGa/Ti/SCD resonators.

**10. Performance comparison between the proposed sensor and previously reported ΔE-effect-based resonant magnetic sensors**

**Table R1.** Comparison performance of ΔE-based resonant magnetic sensor ^[1]^.

| Materials | Structure | Resonance frequency (kHz) | Q factor | Sensitivity  (Hz/mT) | Limitation of detection (nT/Hz^1/2^) | Ref. |
| --- | --- | --- | --- | --- | --- | --- |
| FeGa/PZT | Cantilever | 12.45 | 1132 | 5.47 |  | ^[2]^ |
| Metglas/PZT | Bridge | 0.55~0.85 |  | 4.7×10^-3^ | 4000 | ^[3]^ |
| FeCoSiB/  AlN/Si | Cantilever | 324 | 230 |  |  | ^[4]^ |
| FeCoSiB/  AlN/Pt/SiO_2_ | Cantilever | 6.4~6.65 | 260 | 100 | 12 | ^[5]^ |
| FeCoSiB/  AlN/Si | Cantilever | 7.6 | 905 |  | 0.14 | ^[6]^ |
| CoFe/PZT | Bridge | ~110.1 | 2728 | <10 | 0.025 | ^[7]^ |
| FeGa/Quartz | (Double-ended tuning fork) DETF | 38.199 | 3318 | 35 |  | ^[8]^ |
| FeGaB/Ti/  SCD | Cantilever | 547.034 | 4376 | 152.1 |  | ^[1]^ |
| FeGa/Ti/SCD | Cantilever | 393.381 | 5462 | 12.7 | 2.74 | This work |

Although several ΔE-effect resonant magnetic sensors exhibit higher sensitivity or lower detection limits under room-temperature conditions, the primary focus of the present work is elevated-temperature operation. Most previously reported ΔE-effect MEMS magnetic sensors have been investigated near room temperature, whereas high-temperature magnetic sensing based on ΔE-effect resonators remains rarely reported. The present SCD platform therefore provides a route toward elevated-temperature resonant magnetic sensing and magnetic-field mapping.

**11. Magnetic sensing performances of the magnetic sensors array with varying position of a magnet**

**Figure S10.** Magnetic sensing performance of the SCD magnetic sensors array. The magnet is translated along the *z*-axis in 1 mm increments, with its initial position set at *z_0_* = 10 mm above the horizontal surface of the magnetic sensor array. **a** Resonance frequency shift with magnetic field at *z*= *z_0_*+1 mm. **b** Resonance frequency shift with magnetic field at *z*= *z_0_*+2 mm.

**12. Magnetic field images of the magnet (rotated 90° clockwise relative to that shown in the main text) acquired by the magnetic sensor array at varying z-axis positions**

**Figure S11.** Magnetic field imaging of the magnet (the magnet referenced in the text, rotated 90° clockwise.) via the magnetic sensor array with changing position of the magnet along *z* coordinate as *z_0_*, *z_0_*+1 mm, and *z_0_*+2 mm. The magnetization direction, *M*, which is along the longitudinal axis of the magnet, rotated together with the magnet.

**13. Magnetic sensitivities of the magnetic sensors array at 573 K**

**Figure S12.** Magnetic sensitivities of the magnetic sensor array measured at 573 K. Each sensing pixel was calibrated at 573 K, and the resulting temperature-specific sensitivities were used for reconstruction of the magnetic-field map at the same temperature.

**14. Photograph and schematic diagram of the experimental setup for magnetic field mapping**

**Figure S13.** Photograph and schematic illustration of the magnetic-field mapping setup.

**References**

[1]	Z. Zhang, L. Sang, J. Huang, W. Chen, L. Wang, Y. Takahashi, S. Mitani, Y. Koide, S. Koizumi, M. Liao, *Carbon* **2020**, 170, 294.

[2]	M. A. Indianto, M. Toda, T. Ono, *Sensors and Actuators A: Physical* **2021**, 331, 112985.

[3]	J. Kiser, P. Finkel, J. Gao, C. Dolabdjian, J. Li, D. Viehland, *Applied Physics Letters* **2013**, 102.

[4]	B. Gojdka, R. Jahns, K. Meurisch, H. Greve, R. Adelung, E. Quandt, R. Knöchel, F. Faupel, *Applied Physics Letters* **2011**, 99.

[5]	R. Jahns, S. Zabel, S. Marauska, B. Gojdka, B. Wagner, R. Knöchel, R. Adelung, F. Faupel, *Applied Physics Letters* **2014**, 105.

[6]	S. Zabel, C. Kirchhof, E. Yarar, D. Meyners, E. Quandt, F. Faupel, *Applied Physics Letters* **2015**, 107.

[7]	M. Staruch, C. Kassner, S. Fackler, I. Takeuchi, K. Bussmann, S. Lofland, C. Dolabdjian, R. Lacomb, P. Finkel, *Applied Physics Letters* **2015**, 107.

[8]	L. Bian, Y. Wen, Y. Wu, P. Li, Z. Wu, Y. Jia, Z. Zhu, *IEEE Transactions on Electron Devices* **2018**, 65, 2585.
